# Supplementary material for: Splice-Junction-Based Mapping of Alternative Isoforms in the Human Proteome
Source: Cell Rep. Author manuscript; Available in PMC 2020 Jan 15. (PMC6961840; doi:10.1016/j.celrep.2019.11.026)
Supplement: 2 [file NIHMS1546469-supplement-2.zip › DF1/PXD009737/TestisLysC/TestisLysC_38_DDX60L_NGWKGSDMLYLMRWLQSFREMTQLILNEMPK.pdf]

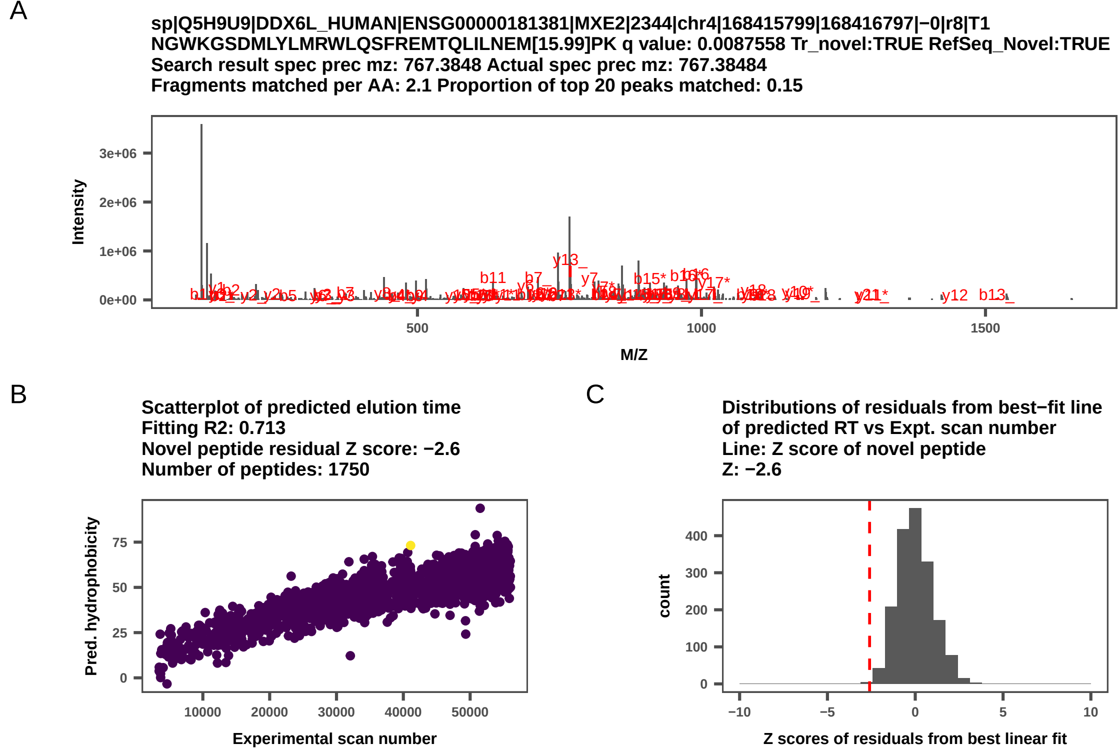
A
sp|Q5H9U9|DDX6L_HUMAN|ENSG00000181381|MXE2|2344|chr4|168415799|168416797|−0|r8|T1
NGWKGSDMLYLMRWLQSFREMTQLILNEM[15.99]PK q value: 0.0087558 Tr_novel:TRUE RefSeq_Novel:TRUE
Search result spec prec mz: 767.3848 Actual spec prec mz: 767.38484
Fragments matched per AA: 2.1 Proportion of top 20 peaks matched: 0.15
Intensity
3e+06
2e+06
1e+06
0e+00
M/Z
500
1000
1500
y13_
b11
b7
y7
b15*
y17*
y12
b13_
B
Scatterplot of predicted elution time
Fitting R2: 0.713
Novel peptide residual Z score: −2.6
Number of peptides: 1750
Pred. hydrophobicity
75
50
25
0
10000
20000
30000
40000
50000
Experimental scan number
C
Distributions of residuals from best−fit line
of predicted RT vs Expt. scan number
Line: Z score of novel peptide
Z: −2.6
count
400
300
200
100
0
−10
−5
0
5
10
Z scores of residuals from best linear fit
